# Supplementary material for: Compact quantum dot models for analog microwave co-simulation
Source: npj Quantum Inf. 2025 Dec 13;11(1):194. doi: 10.1038/s41534-025-01140-8 (PMC12714576; doi:10.1038/s41534-025-01140-8)
Supplement: Supplementary file 1 — Supplementary Information [file 41534_2025_1140_MOESM1_ESM.pdf]

Supplementary Material to: Compact Quantum Dot Models for Analog Microwave co-Simulation

Lorenzo Peri,[1,2,*] Alberto Gomez-Saiz,[1,3] Christopher J. B. Ford,[2] and M. Fernando Gonzalez-Zalba[1,4,5,†]

[1] *Quantum Motion, 9 Sterling Way, London, N7 9HJ, United Kingdom*
[2] *Cavendish Laboratory, University of Cambridge, JJ Thomson Ave, Cambridge CB3 0HE, United Kingdom*
[3] *Department of Electrical and Electronic Engineering, Imperial College London, London SW7 2AZ, United Kingdom*
[4] *CIC nanoGUNE BRTA, 20018 Donostia-San Sebastian, Basque Country, Spain*
[5] *IKERBASQUE, Basque Foundation for Science, 48013 Bilbao, Basque Country, Spain*

(Dated: September 8, 2025)

SM1. CONSIDERATIONS ON PERFORMANCE AND SCALABILITY

Here, we discuss the scaling of the model with respect to the size and number of quantum elements included in a simulation circuit. Firstly, we discuss the scaling properties with respect to the number of quantum levels one intends to simulate. To fully describe a system with N levels, the density matrix ρ takes the form of a $N \times N$ self-adjoint matrix with unitary trace. Therefore, it contains $N^2 - 1$ (real) degrees of freedom to be evolved within the model (i.e. $N^2 - 1$ copies of the nets described in Fig. 2b). This polynomial scaling is a traditional issue of large quantum simulations [S1, S2], and in a way the fundamental reason why quantum computing is technologically interesting in the first place [S3]. Because of this, typical caveats and considerations apply: high-energy states that are seldom occupied may be neglected as they do not contribute significantly to the dynamics (this also aids convergence as it eliminates fastly oscillating terms in the dynamics of the coherences), and weakly coupled quantum sub-systems could be evolved separately, with their interaction treated perturbatively. This is facilitated by the netlist structure of electrical simulations, where certain weak couplings may be modelled as a classical link between separate quantum models (i.e. electrostatic or magnetostatic couplings may be replaced by suitable mutual capacitances or inductances).

However, we must make clear that the formalism discussed in this work does *not* suggest using analog circuit simulators as an efficient quantum simulators (e.g. for running quantum algorithms). Instead, the value of the proposed formalism is providing a tool for exploring the interplay between classical and quantum information, and between classical and quantum hardware. Our models enable a quantum hardware designer to leverage well-established EDA design tools for quantum circuit development, while unlocking quantum devices as another element in the design palette available to cryogenic analogue circuit designers.

* lp586@cam.ac.uk
† fernando@quantummotion.tech

Nonetheless, we must point out that modern circuit simulators *are* extremely efficient non-linear coupled, differential algebraic equations solvers [S4], capable of handling complex circuits with over a million components. Thus, while the full simulation of utility-scale quantum processing units (QPUs) with hundreds of thousands, if not millions of physical qubits [S5] may be out of the reach of (any) classical simulation, we see great value (and treatable computational complexity) in the co-simulation of small functional subsets of a QPU and the interfacing electronics. For example, a functional simulation of a subset of qubits, sensors and readout electronics may be used to optimize the readout chain [S6], while a functional simulation of a subset of qubits and control electronics may be used to assess the impact of non-idealities in signal generation of qubit operation [S7]. Quantum error correction, with the need for fast interaction between quantum and classical elements in a tiled manner [S8], may also be a fertile ground that could benefit from the developed co-simulation framework.

Furthermore, leveraging EDA tools also allows the simulation of quantum systems to leverage the plethora of extremely optimized analyses that are traditionally not available in bespoke quantum simulators, but that constitute the backbone of analogue circuit simulations. This ranges from simple small-signal analyses to more complex and specialized ones.

To substantiate our claims and provided quantitative data, we use the circuit in Fig. S1 as a benchmark of the co-simulation computational cost. The circuit is composed of a device under test (DUT), behaving as a non-linear impedance, with a bias-tee connected to the left terminal, and an RLC resonator on the right terminal. The bias tee and the resonator are connected to ac lines connected to $50\,\Omega$ port respectively. The resonator has an integrated RC bias network. The DUT is populated either with a DQD charge qubit (blue square), a DQD singlet-triplet spin qubit (red triangle)—modeling of 2 and 5 quantum levels respectively—or a MOSFET using the BSIM-IMG model version 102.9.6 [S9] (black pentagon) which serves as a performance baseline. The MOSFET front gate is connected to the bias-tee, and the source and drain are tied together and connected to the resonator, in what is know as inversion-mode MOS varactor [S10]. The MOS-

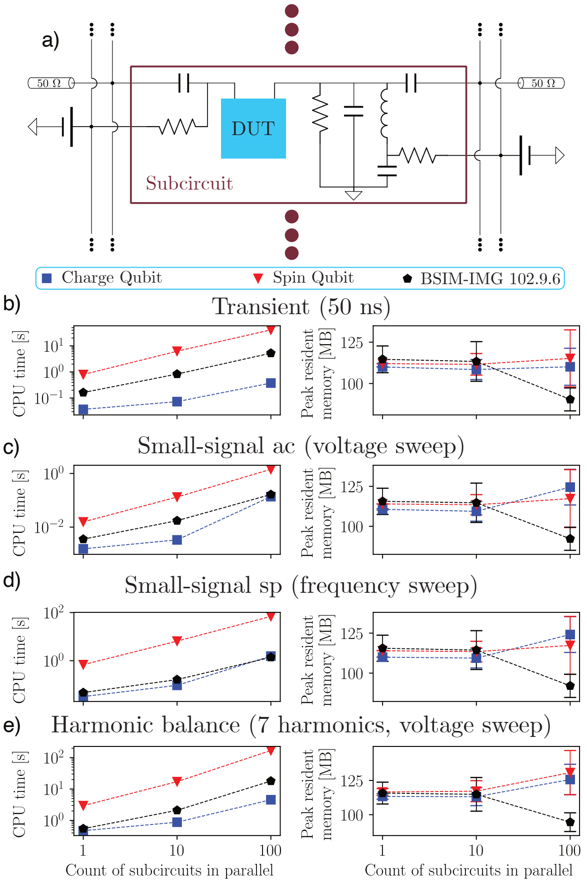

FIG. S1. **Performance of co-simulation.** (a) Device under test (DUT). The DUT is populated either with a DQD charge qubit (blue square), a DQD singlet-triplet spin qubit (red triangle), and a BSIM-IMG MOSFET [S9] model vesion 102.9.6 (black pentagon) as a reference. Parallel instances of subcircuit (within the red box) are then instantiated 1, 10, and 100 times to evaluate how the computational cost scales. The analysis ran are (b) a 50 ns transient, (c) a small-signal circuit domain (ac) analysis with a 100-point sweep of the dc gate voltages, (d) a small-signal network domain (sp) analysis sweeping the excitation frequency around the RLC resonance , and (e) a 7-harmonic harmonic balance large-signal (hb) analysis, with a 100-point sweep of the dc gate voltages. We perform 25 runs for each test, presenting average CPU time (left) and peak resident memory (right). The error bars represent one standard deviation (omitted on the left for legibility as significantly smaller than marker size ($\lesssim 1\%$)). All simulations are performed on a single core, on an Intel®Xeon®Platinum 8375C CPU @ 2.90GHz with 256 GB memory.

FET back gate is grounded. Parallel instances of the subcircuit (within the red box) are then instantiated 1, 10, and 100 times to evaluate how the computational cost scales. The analysis ran are a 50 ns transient (panel b), a small-signal circuit domain (ac) analysis with a 100-point sweep of the dc gate voltages (panel c), a small-signal network domain (S-parameter, sp) analysis sweeping the excitation frequency around the RLC resonance (panel d), and a 7-harmonic harmonic balance large-signal (hb) analysis, with a 100-point sweep of the dc gate voltages (panel e). We perform 25 runs for each test, presenting average CPU time (left) and peak resident memory (right). All simulations are performed on a single core, on an Intel®Xeon®Platinum 8375C CPU @ 2.90GHz with 256 GB RAM memory. We would like to stress that our models are optimized for accuracy and code legibility, not for simulation performance. The following results thus ought to be taken as a baseline that can certainly be improved (particularly via the use of accelerated linear algebra libraries, natively lacking in standard Verilog-A). Figure Fig. S1b-e show the simulation CPU times and memory usage for the three circuit variants. Memory usage is comparable across all models, and the charge qubit shows comparable simulation times to the BSIM-IMG model. There is an approximate order of magnitude difference between charge ($N = 2$) and singlet-triplet spin ($N = 5$) qubits, reflecting the increase in the number of internal nets. We speculate, however, that the presence of the non-avoided singlet-$|T_-\rangle$ level crossing in the spin qubit is impacting the simulation run time. The change of ground state between singlet and triplet is causing significantly different thermal equilibrium populations within and outside the PAuli-blockaded region, potentially causing convergence issues. Moreover, the small energy differences (e.g. within the triplet manifold at low magnetic fields or around non-avoided crossings) require significant care in the evaluation of the Bose-Einstein distribution in order to avoid numerical issues. Contrarily, this is not a concern in the gapped charge qubit due to the avoided crossing (generally large with respect to thermal energy). Therefore, if performance was to become a bottleneck, significant improvements can undoubtedly be achieved (for example, the spin qubit Lindbladian has a large number of common sub-expressions that may be cached, and a hard cutoff for evaluating exponential functions, rather than the Verilog-A native function `$limexp` [S4], may be implemented)

[S1] B. Fauseweh, Nature Communications **15**, 10.1038/s41467-024-46402-9 (2024).
[S2] O. Morsch, G. M. Palma, and D. Rossini, La Rivista del Nuovo Cimento **48**, 275–313 (2025).
[S3] J. Preskill, Quantum computing 40 years later (2023), arXiv:2106.10522 [quant-ph].
[S4] C. C. McAndrew, G. J. Coram, K. K. Gullapalli, J. R. Jones, L. W. Nagel, A. S. Roy, J. Roychowdhury, A. J. Scholten, G. D. J. Smit, X. Wang, and S. Yoshitomi, IEEE Journal of the Electron Devices Society **3**, 383–396 (2015).
[S5] M. F. Gonzalez-Zalba, S. De Franceschi, E. Charbon, T. Meunier, M. Vinet, and A. S. Dzurak, Nature Electronics **4**, 872–884 (2021).

[S6] G. Kiene, O. Pietx-Casas, M. Babaie, L. M. K. Vandersypen, and F. Sebastiano, IEEE Transactions on Circuits and Systems I: Regular Papers , 1 (2025).

[S7] J. van Dijk, E. Kawakami, R. Schouten, M. Veldhorst, L. Vandersypen, M. Babaie, E. Charbon, and F. Sebastiano, Physical Review Applied **12**, 044054 (2019).

[S8] B. Branchini, D. Conficconi, D. Sciuto, and M. D. Santambrogio, in *2023 IEEE International Conference on Quantum Computing and Engineering (QCE)*, Vol. 2 (IEEE, 2023) pp. 338–339.

[S9] S. Khandelwal, Y. S. Chauhan, D. D. Lu, S. Venugopalan, M. Ahosan Ul Karim, A. B. Sachid, B.-Y. Nguyen, O. Rozeau, O. Faynot, A. M. Niknejad, and C. C. Hu, IEEE Transactions on Electron Devices **59**, 2019–2026 (2012).

[S10] P. Andreani and S. Mattisson, IEEE Journal of Solid-State Circuits **35**, 905 (2000).
